# Staff Perceptions of Compassionate Care Visits for Nursing Home Residents During Restricted Visitation

**DOI:** 10.1155/jonm/4128918

**Published:** 2024-11-28

**Authors:** Melissa McClean, Kalei Kowalchik, Jacqueline Mogle, Liza Behrens, Joan G. Carpenter

**Affiliations:** ^1^Organizational Systems and Adult Health Department, University of Maryland School of Nursing, Baltimore, Maryland, USA; ^2^The Ross and Carol Nese College of Nursing, The Pennsylvania State University, University Park, Pennsylvania, USA; ^3^Department of Psychology, Clemson University, Clemson, South Carolina, USA

**Keywords:** end-of-life, infectious outbreaks, restrictions, risk perceptions, social isolation, visitation

## Abstract

**Aim:** To describe compassionate care (CC) visits during visitation restrictions from the perspective of nursing home (NH) staff.

**Background:** During the COVID-19 pandemic, guidance was implemented to restrict visitation in NH communities to protect both residents and staff from risk of infectious outbreak. As a result, many NHs instituted “CC visits” to allow visitation for vulnerable residents. However, it is unclear how CC visits were defined, operationalized, and how their associated benefits and risks were perceived by staff.

**Methods:** We conducted an exploratory qualitative descriptive study using semistructured interviews and analyzed data using directed content analysis among NH direct care staff.

**Results:** From June 2021 through March 2022, we recruited nursing, social work, and activities NH staff participants (*N* = 24). Most were female (88%), White (79%), and had more than 3 years of experience working in NHs (79%). We found three main thematic categories and associated subthemes: (1) *Appropriate Use of CC Visits* (visits for residents experiencing imminent death, to support resident's psychosocial needs, and for family members exhibiting concern for the resident); (2) *Benefits of CC Visits* (resident and staff physical and psychosocial benefits); and (3) *Risks of CC Visits* (resident and staff risks of facilitating visits and contracting illness). In this study, benefits of CC visits outweighed risks.

**Conclusions:** In this study, we describe how NH staff describe CC visits, their use during the COVID-19 pandemic, and associated risks and benefits. This includes when visitation could be provided to residents and the impact it had on the resident's well-being.

**Implications:** This study highlights the need to consider the physical and psychosocial consequences of social isolation of NH residents.

**Impact:** Study findings can be used to provide guidance in future public health emergencies when visitation restrictions are instituted.

**Reporting Method:** We have adhered to the Standards for Reporting Qualitative Research (SRQR) guidelines.

**Patient or Public Contribution:** Direct care NH staff including social workers (SWs), nursing staff, and activity directors between the ages of 18 and 89 years old were English speaking and willing to participate in a semistructured interview outside of work hours participated in this research. Potential participants contacted members of the study team by phone or email to express their interest in study participation. After confirming eligibility and willingness to participate, an email link was sent to participants with the electronic information/consent sheet using the Qualtrics web-based survey platform (Qualtrics, Provo, Utah). Once participants consented to participate, they were directed to an electronic demographic questionnaire and risk perception survey. At the end of the risk perception survey, participants could indicate if they were willing to be contacted for a semistructured interview to discuss restricted social activities and visitation mandates in NHs. Interviews were scheduled via Zoom video conference during a time outside of work hours. Following participant verbal consent, interviews were audio-recorded. Participants were given a $30 electronic gift card.

**Summary**

  What does this paper contribute to the wider global clinical community?
• Nursing home (NH) staff perceive the benefits of compassionate care (CC) visits during visitation restriction outweigh the risks.
• During times of visitation restriction, NH staff use CC visits for residents experiencing imminent death, to support resident's psychosocial needs, and for family members exhibiting concern for the resident.

## 1. Introduction

Older adults in congregate living settings experience greater health risks during infectious outbreaks [1]. Infection control mandates are intended to protect the 1.3 million frail and medically complex individuals residing in the United States NH communities [2]. However, these mandates can do more than inhibit viral or bacterial transmission. For example, during the COVID-19 pandemic, health officials issued temporary measures restricting visitors in NH communities to prevent and minimize the spread of the virus that caused COVID-19 infection [3–6]. As a result, nonessential healthcare personnel and unpaid family caregivers were banned from entering NHs which resulted in the physical and social isolation of residents.

Physical and social isolation is not only in direct contrast to what residents prefer and expect in their personal living setting, but it is also in contrast to how Centers for Medicare and Medicaid Services (CMS) intends care to be delivered to medicare and medicaid beneficiaries residing in NH communities [7]. By initiating visitor restrictions and limiting social activities, NH staff members were prevented from providing residents with person-centered care, an approach to care focused on an individual's preferences and interdisciplinary goals [8]. Residents, many of whom experience mild to severe cognitive impairment, were isolated and NH staff members were unable to honor resident and person-centered preferences such as communal dining, group social activities, and close physical contact.

Beginning in May 2020 and throughout the following year, CMS issued reopening recommendations to expand visitation in certain situations and subsequently provided periodic updates to the guidance as new information was available [3]. Notable additions to the guidance during this time were examples of “CC” situations or times when CC visits were allowed. CC visits, at times used synonymously with “comfort care” and understood as an exception only in end-of-life situations, were permitted when residents exhibited physical and cognitive decline and emotional distress. The frequency of CC visits in NHs depended on many risk factors including the rates of infection within the NH, the ability to control infection with mitigation strategies (e.g., vaccine availability and adequate personal protective equipment supply), ample outdoor and indoor space for visitation, and sufficient staffing to arrange and schedule visitors [9].

Based on the anecdotal experiences of our research team members who practiced in NHs during the pandemic, interpretation of the guidance and the concept of CC visits was subject to broad interpretation. Some NH staff reserved CC visits for residents imminently dying and on hospice care. Others made decisions to allow CC visits strictly based on information related to state or local infection rates. While the use of the term “CC” is a recognizable nomenclature in CMS guidance on visitation in NH during the COVID-19 pandemic, it had nonspecific meaning prior to the pandemic. During an extensive search, we were unable to find the empirical literature supporting a universal definition of CC visits or CC visitation. To address this gap in the literature, we aimed to describe how NH staff perceived the use of CC visits in NHs during the COVID-19 pandemic.

## 2. Methods

This exploratory qualitative descriptive study was part of a larger study designed to improve understanding of direct care (e.g., frontline) NH staff risk perceptions and experiences associated with nursing operations and culture of care during times of restricted visitation and activities (e.g., communal dining, social engagement, walking in hallways, and visitors during illness) [10]. The research team recruited, enrolled, and collected semistructured interview data over a 10-month period between June 2021 and March 2022. Direct care NH staff participants were identified from social media, professional group discussion boards, Listservs, and university-sponsored study websites. We also shared flyers with NH contacts and colleagues of our study team. This study was approved by the Institutional Review Boards at The University of Maryland, Baltimore, and The Pennsylvania State University. Study team members were nurses, several of which who cared for residents in NHs during restricted visitation. The team practiced reflexivity through weekly team meetings to address experiences that may have influenced data collection and analysis [11].

### 2.1. Sample

The research team used purposive sampling to identify interdisciplinary, direct care NH staff including social workers (SWs), nursing staff, and activity directors [12]. Participants met inclusion criteria if they were currently working or had worked in a NH during the COVID-19 pandemic, were between the ages of 18 and 89 years, were English speaking, and willing to participate in a semistructured interview outside of work hours. A total of 24 NH staff participated in interviews. The sample of NH staff included representation from activities staff, certified nursing assistants (CNA), licensed practical nurses (LPN), registered nurses (RN), and SWs.

### 2.2. Data Collection and Procedures

Potential participants contacted members of the study team by phone or email to express their interest in study participation. After confirming eligibility and willingness to participate, an email link was sent to participants with the electronic information/consent sheet using the Qualtrics web-based survey platform (Qualtrics, Provo, Utah). Once participants consented to participate, they were directed to an electronic demographic questionnaire and risk perception survey for the larger study [13]. All participants had email access, and none requested to complete the instruments via phone. The team used descriptive statistics to summarize the demographic data through SPSS V.29.

**Figure 1:**
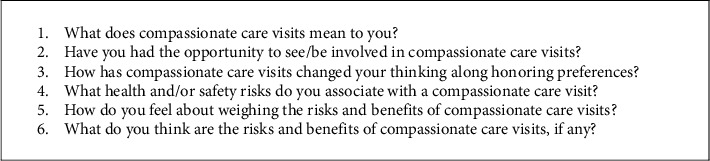
Sample semistructured interview questions.

At the end of the risk perception survey, participants could indicate if they were willing to be contacted (by phone or email) for a semistructured interview to further discuss restricted social activities and visitation mandates in NHs. Following participant verbal consent, interviews were scheduled via Zoom video conference during a time outside of work hours. We used a semistructured interview guide that asked about positive and negative risk perceptions, and benefits and challenges of CC visitation (Figure 1). Interviews were audio-recorded. Participants who completed both the survey and interview were given a $30 electronic gift card.

Deidentified interview transcripts were organized, stored, and analyzed in QSR International's NVivo 12 software for Windows/Mac according to participant code/pseudonym (QSR International Pty Ltd., 2018). Interviews were transcribed verbatim and anonymized by trained study staff with nonverbal pauses and emotional expressions (i.e., laughing and crying) retained in the transcript. Three study team members used two transcripts to create a codebook. The codebook was refined based on the team feedback throughout the analysis. The study team applied codes to the remaining transcripts. Coding disagreements were resolved by consensus.

### 2.3. Data Analysis

For this analysis, the team used deductive thematic content analysis [14] to identify emerging thematic categories in the data that applied to our research question: How did NH staff perceive the use of CC visits during times of visitation restriction? This pre-existing question and the interview guide align with the preference-based person-centered risk engagement model [15] and guided the team in exploring concepts such as visitation restrictions and the risks and benefits of CC visits. An iterative process detailed in an audit trail allowed for examination and re-examination of coded data for accuracy and meaning.

## 3. Results

Participants' (*n* = 24) age ranged from 18 to 62 years (*M* = 39, SD = 8.31); most identified as females (*n* = 21). Participants identified as White or Caucasian (*n* = 19), Black or African American (*n* = 3), or Asian or Pacific Islander (*n* = 2). Seventy-seven percent of survey participants who completed the survey (*n* = 31) returned for an interview. Most participants (*n* = 19) reported more than 3 years of experience working in a NH setting, and all received training on person-centered care prior to their interview. Participants' demographic information can be found in Table 1. Directed content analysis revealed three thematic categories: (1) staff determining appropriate use of CC visits, (2) staff perceptions of benefits of CC visits, and (3) staff perceptions of risks of CC visits. Each thematic category includes subcategories (Table 2 Thematic Table).

### 3.1. Staff Determining Appropriate Use of CC Visits

Within this thematic category, we found an evolving narrative. Before the COVID-19 pandemic, delivering “CC” was reserved for those residents imminently dying. One activities staff member stated, “CC visits have always meant end-of-life until COVID came” (Survey Participant ID_2). Our data illustrate participants delivering a more holistic and resident-centered care to residents with physical, social, and emotional needs, as well as using CC visits for family members exhibiting concern for their loved ones. As a result, staff made decisions determining appropriate use of CC visits. Therefore, our data aligned with three distinct subcategories: (1) visits for residents experiencing imminent death, (2) visits to support resident's psychosocial needs, and (3) visits for family members exhibiting concern for the resident. Individuals involved in CC included both nonstaff visitors (e.g., a family member or friend of the resident or an individual identified as a “compassionate caregiver”) and interdisciplinary NH staff members (e.g., nursing and activities staff and chaplains).

#### 3.1.1. CC Visits for Residents Experiencing Imminent Death

During interviews, participants were asked to describe what CC visits meant or how they were executing CC visits in their NH community. One SW defined a CC visit by stating “To me, it means that families should be able to see their loved ones before they declined fully” (Participant ID_9). Several staff explained CC visits would be implemented in a resident's care plan along with palliative or end-of-life care or when residents experienced a transition or decline in health to necessitate a referral to hospice. One APRN provided an example of how CC visits were integrated in the NH community by saying, “If there was a patient that was on the verge of passing away, maybe going on hospice, we would allow one family member to come for compassionate visits. I think it's good for the residents to have someone there with them. I'm sure they're frightened, scared, and not understanding what's going on with their health… a CC visit would be allowing someone to come in when a resident patient is deteriorating” (Participant ID_7).

#### 3.1.2. CC Visits to Support Resident's Psychosocial Needs

Participants also discussed using CC visits for residents exhibiting a need for emotional support. A SW described a CC visit as, “…not necessarily an end-of-life visit or a hospice visit. It can be just an emotional support visit for somebody that's having a hard time” (Participant ID_9). Also, when a resident exhibited changes in behavior, staff members were prompted to arrange a CC visit. Another SW explained, “…if we had people that has increased wandering or if they had to score higher on their depression inventory, suicidal ideation, anybody that went on hospice services… if there was a recent death in the family, just any kind of life changing event… acting out whether it was yelling at people or crying… then we were looking at CC visits” (Participant ID_37).

#### 3.1.3. CC Visits for Family Members Exhibiting Concern for the Resident

Participants also described CC visits as a modality for alleviating the worry or concern of a family member. For example, an activities staff member stated, “…if a resident wanted to see their family, we would definitely make that happen. But 99.9% of the times it's the family screaming ‘I haven't seen my dad all week!'…and we—we want to make that happen. So, I not only honor the residents, we do the best we can [to] honor the family members too” (Participant ID_3). This example illustrates that NH staff provided a family-centered level of care where compassion, as a virtue and not just a care modality, extends to others beyond the resident needing care. Furthermore, staff reported assessing and addressing the well-being of residents and family alike. For example, one SW stated, “Not only were the residents declining, but their family members were as well when they couldn't see them. So we actually ended up putting a lot of our residents on CC that were…teetering on meeting the qualifications for CC. But we…exaggerated some things so that family were able to come in and be with them” (Participant ID_35).

### 3.2. Staff Perceptions of Benefits of CC Visits

Our data described a bidirectional relationship between the benefits and risks of CC visitation, such that the benefits overwhelmingly outweighed the risks and that challenges were overcome to create more benefit for the resident, staff, and family. In addition, staff described CC visitation as a positive strategy to provide comfort to residents receiving hospice care and specifically addressed feelings of loneliness and isolation in residents who are experiencing a decline in their physical health or psychological well-being. An activities staff member described what populations would benefit from CC visitation by stating, “…Especially people who are isolated in their rooms, who have declined in health, it could be physical health, or it could be mental health. They are most definitely in need of care and of having people spend some time with them and helping them where they're at” (Participant ID_38).

#### 3.2.1. Resident Benefits of CC Visits

Most staff members disclosed that when residents participated in a CC visit, it positively impacted the resident's physical and psychosocial well-being. Staff also discussed improvement in residents' mood when they reunited residents with their family members. One CNA stated, “The benefits of it [CC visits] are the well-being of the residents. Makes them happy, makes them engaged in their life, and not feel so powerless” (Participant ID_4). Staff emphasized that the integration of CC visits had a positive impact on residents' well-being. A RN stated, “The benefits overall are good for their psychosocial wellbeing. It's good for their physical well-being. They tend to be more active when they have more visitors, and they tend to be better cared for when they have family members that are actively involved in their care” (Participant ID_40).

#### 3.2.2. Staff Benefits of CC Visits

Staff described CC visits as beneficial not just to the resident but the staff as well. When limited visitation was permitted after the first few months of the COVID-19 pandemic, the experience of having outside visitors in the NH community was positive and emotional for both staff and residents. A LPN stated, “I know the first time when we had one of our residents' moms…come into our facility. She was the first visitor we allowed back. Everybody cried. Everybody cried…it was just … so good to see someone else in our building” (Participant ID_5). Another example of staff benefit is the following quote from one activities staff member that stated, “I love seeing the smiles on their faces when they can do something, like get out…” (Participant ID_10). Also, one SW states, “… I can tell you whenever the compassionate caregiving visits started, we were very happy that we were allowed to be able to do that…” (Participant ID_15). These statements illustrate that staff experienced positive emotions when residents were able to enjoy activities. While CC visitation poses some risk of transmission of COVID-19, several staff found that the benefit to residents, families, and staff outweighed potential risks and challenges they experienced.

### 3.3. Staff Perceptions of Risks of CC Visits

The primary risk of CC visits staff identified was the risk of the resident contracting an illness such as COVID-19 from someone outside of the NH (e.g., family member or NH staff). And while contracting COVID-19 is an identified risk of CC visitation, some NH staff members felt that the risk of contracting COVID-19 was equivalent to the risk of not utilizing CC visits for affected NH residents. For example, one CNA stated, “…there's always going to be a risk, but I think it's one of the risks that you have to take. I think visitors [are] a big part of someone's mental…health, and they're very positive” (Participant ID_18).

#### 3.3.1. Resident Risks of CC Visits

Staff discussed risks of facilitating CC visits within the NH community during COVID-19. One CNA stated, “It's definitely made that [CC visits] more conflicting because you just see the benefits and how much it means to them [the resident], and you have to remind yourself that yes, their safety is important, even though you can see how much it means to them personally and emotionally. You have to always have in the back of your mind that we're in the middle of a pandemic, and sometimes their wishes can't be honored because of that. It does make it very conflicting ‘cause you'd want to provide the best care for them and make them happy, but sometimes you can't do that just for their overall health, so it can be pretty difficult” (Participant ID_13). In addition, one RN stated, “Hospice brought COVID in…and she, the director of nursing…wanted to end hospice visits altogether” (Participant ID_23). Staff described that CC visits not only raised the potential for risk but also the actual occurrence of infectious outbreak.

#### 3.3.2. Staff Risks of CC Visits

While staff identified that NH residents are at risk of contracting COVID-19 due to CC visits, they report that *other care staff* throughout the NH may also be exposed to illness. NH staff discussed that if staff or families do not adhere to safety precautions during their visits (i.e., not wearing personal protective equipment appropriately, not washing hands, and not maintaining a safe distance), it poses a higher risk to the NH community. One LPN described, “It [CC visits] puts your staff at a higher risk. But then you know we can encourage staff to wear a mask and staff to do hygiene and staff not to go to concerts and large groups and things. But we can't control their outside life either” (Participant ID_5). One NH facility that allowed CC visits experience the negative consequence of an infectious outbreak. A SW stated, “…3 days after a CC visit on one of my unit, we had an outbreak of COVID on that unit and that really made me personally question it” (Participant ID_10).

## 4. Discussion

Infectious outbreaks and associated infection control measures are common in NH communities [1]. The COVID-19 pandemic prompted strict infection control mandates, and therefore, NH staff experienced challenges in honoring resident preferences and delivering person-centered care. Furthermore, decreased visitation from essential family members who acted as care partners created negative consequences for people living with serious illness [16]. Consequences included social isolation, decreased oral feeding for people living with dementia, increased pain, a greater need for help with activities of daily living, and family member's need for more communication from staff about a resident's needs [17].

The results of our study indicate that NH staff made decisions to determine appropriate use of CC visits. While health officials provided as much guidance as possible, NH staff factored their resources, knowledge of gerontology, and clinical judgment in their decision-making process. Regardless, participants of the study described the process as difficult. The recent research indicates that decision-making in stressful work environments negatively impacts NH staff contributing to alarming rates of moral and ethical distress and compassion fatigue [18]. These findings highlight the importance occupational support in times of crisis and efforts to strengthen the NH workforce.

When NH staff limited visitation of family/care partners due to guidance mandates, NH staff reported that residents suffered physically, spiritually, and emotionally. This finding supports the empirical literature; Savage et al. reported that long-term NH residents without family or friend contact experienced an increase of close to 60% greater excess mortality [19]. NH staff perceived improved physical and emotional well-being as benefits of CC visits and supported their use when possible as benefits were thought to outweigh the risk of COVID-19 transmission. Also, participants perceived less risk of COVID-19 transmission when adequate safety precautions were in place. Our findings suggest the need to expediently assess the risk/benefit profile of CC visits and viral transmission during restricted visitation to avoid the detrimental impact of prolonged social isolation in NHs.

Lastly, when infection control and risk mitigation are of utmost importance, honoring residents' holistic needs and preferences through CC is tenuous [20]. The literature published prior to the pandemic defined CC as adjusting to the range of a patient's needs effectively and appropriately to provide to high-quality, resident-centered care [21, 22]. The provision of care deemed “compassionate” should occur in all healthcare settings and situations, and especially in NH communities where older and potentially vulnerable adults with serious illness reside. Our findings demonstrate a NH workforce balancing effective infection control practices with care that is safe, earnest, and dignified.

### 4.1. Limitations

We acknowledge several limitations of this study. First, we used social media and professional organizations for recruitment. Not only did participants require personal social media accounts or paid professional organizational membership to benefit from this outreach, but they were also required to have access to a phone, the Internet, and email to communicate with investigators. While our goal was to reach a national sample, we recognize that only direct care workers who were able to contact us and wanted to talk about their experiences enrolled. Missing from this study are those staff who may have suffered moral distress, did not want to talk about their experience, and/or left the profession after being a frontline worker during the pandemic. We also acknowledge the lack of diversity in our sample despite efforts to reach a wide range of direct care worker groups. Lastly, during this study, we were unable to determine whether other factors such as geographic location of the NH staffs' workplace and local rates of COVID-19 transmission, morbidity, and mortality may have influenced risk perception of CC visits. Despite these limitations, this work is important for leaders during times of uncertainty that require restriction in resident's social activities and visitation.

## 5. Conclusion

In this study, we describe staffs' perceptions of visitation restrictions and the use of CC visits during the COVID-19 pandemic. Overwhelmingly, staff perceived the benefits of CC visits during visitation restriction outweigh the risks. CC visits were shown to be critical in supporting the physical and psychological well-being of NH residents who experienced decline due to social isolation. Study findings can be used to provide guidance in future public health emergencies when visitation restrictions are instituted. NH staff members are charged with expediently assessing the risk-benefit profile to avoid the risk of physical decline due to the psychoemotional harms of isolation. To do so, NHs require equitable access to personal protective equipment such as face masks or shields, garment protection, and disinfectants. The vitality and well-being of residents, staff, and care partners in vulnerable congregate living settings requires a unified and coordinated approach from clinicians, administrators, government officials, and our nation's leaders. The costs of restricted visitation are too high to ignore in future infectious outbreaks.

## Figures and Tables

**Table 1:** Participant demographics (*n* = 24).

Age (years)	*M* = 39 (range 18–62)

Gender	Male *n* = 3 (13%)
	Female *n* = 21 (87%)

Race	Black or African American *n* = 3 (13%)
	Caucasian *n* = 19 (79%)
	Asian or Pacific Islander *n* = 2 (8%)

Staff roles	Activities director/staff *n* = 6 (25%), certified nursing assistant *n* = 7 (29%), licensed practical nurse *n* = 1 (4%), and registered nurse *n* = 4 (17%)
	Social worker *n* = 6 (25%)

Education	Less than high school *n* = 1 (4%)
	High school diploma or GED *n* = 5 (21%) and technical school or specialty diploma *n* = 1 (4%)
	Associate degree *n* = 4 (17%), Bachelor's degree *n* = 7 (29%), and Master's degree *n* = 6 (25%)

Years of experience working in nursing home	Less than 1 year *n* = 2 (8%)
	1–5 years *n* = 7 (29%)
	6–15 years *n* = 9 (38%)
	16–20 years *n* = 2 (8%)
	More than 21 years *n* = 4 (17%)

**Table 2:** Thematic table of compassionate care (CC) visits.

Thematic category	Subcategory	Exemplar quote
Staff determining appropriate use of CC visits	CC visits for residents experiencing imminent death	“If there was a patient that was on the verge of passing away, maybe going on hospice, we would allow one family member to come for compassionate visits. I think it's good for the residents to have someone there with them. I'm sure they're frightened, scared, and not understanding what's going on with their health… a compassionate care visit would be allowing someone to come in when a resident patient is deteriorating” (Participant 20)
	CC visits to support resident's psychosocial needs	“…if we had people that has increased wandering or if they had to score higher on their depression inventory, suicidal ideation, anybody that went on hospice services… if there was a recent death in the family, just any kind of life changing event… acting out whether it was yelling at people or crying… then we were looking at compassionate care visits” (Participant 19)
	CC visits for family members exhibiting concern for the resident	“…if a resident wanted to see their family, we would definitely make that happen. But 99.9% of the times it's the family screaming “I haven't seen my dad all week!” …and we—we want to make that happen. So, I not only honor the residents, we do the best we can [to] honor the family members too” (Participant 2)

Staff perceptions of benefits of CC visits	Resident benefits of CC visits	“The benefits overall are good for their psychosocial wellbeing. It's good for their physical well-being. They tend to be more active when they have more visitors, and they tend to be better cared for when they have family members that are actively involved in their care” (Participant 21)
	Staff benefits of CC visits	“I know the first time when we had one of our residents' moms…come into our facility. She was the first visitor we allowed back. Everybody cried. Everybody cried, I mean, it was just … so good to see someone else in our building” (Participant 4)
	Resident risks of CC visits	“It's definitely made that [compassionate care visits] more conflicting because you just see the benefits and how much it means to them [the resident], and you have to remind yourself that yes, their safety is important, even though you can see how much it means to them personally and emotionally. You have to always have in the back of your mind that we're in the middle of a pandemic, and sometimes their wishes can't be honored because of that. It does make it very conflicting “cause you'd want to provide the best care for them and make them happy, but sometimes you can't do that just for their overall health, so it can be pretty difficult” (Participant 14)
	Staff risks of CC visits	“It [compassionate care visits] puts your staff at a higher risk. But then you know we can encourage staff to wear a mask and staff to do hygiene and staff not to go to concerts and large groups and things. But we can't control their outside life either” (Participant 4)

## Data Availability

The data that support the findings of this study are available from the corresponding author on request. The data are not publicly available due to privacy or ethical restrictions.

## References

[1] Centers for Disease Control and Prevention (2020). Serious Infections and Outbreaks Occurring in LTCFs. https://www.cdc.gov/longtermcare/staff/report-publications.html.

[2] Centers for Disease Control and Prevention (2022). Nursing Home Care. https://www.cdc.gov/nchs/fastats/nursing-home-care.htm.

[3] Centers for Medicare & Medicaid Services (2021). CMS Updates Nursing Home Guidance With Revised Visitation Recommendations. *Fact Sheet*.

[4] Centers for Medicare & Medicaid Services (2023). Guidance for Infection Control and Prevention of Coronavirus Disease 2019 (COVID-19) in Nursing Homes (REVISED). https://www.cms.gov/medicareprovider-enrollment-and-certificationsurveycertificationgeninfopolicy-and/guidance-infection-control-and-prevention-coronavirus-disease-2019-covid-19-nursing-homes-revised.

[5] Iaboni A., Cockburn A., Marcil M. (2020). Achieving Safe, Effective, and Compassionate Quarantine or Isolation of Older Adults With Dementia in Nursing Homes. *American Journal of Geriatric Psychiatry*.

[6] Levere M., Rowan P., Wysocki A. (2021). The Adverse Effects of the COVID-19 Pandemic on Nursing Home Resident Well-Being. *Journal of the American Medical Directors Association*.

[7] Centers for Medicare & Medicaid Services (2016). CMS Finalizes Improvements in Care, Safety, and Consumer Protections for Long-Term Care Facility Residents. *Press Release*.

[8] Morgan J. C., Ahmad W., Chen Y. Z., Burgess E. O. (2023). The Impact of COVID-19 on the Person-Centered Care Practices in Nursing Homes. *Journal of Applied Gerontology*.

[9] Centers for Medicare & Medicaid Services (2020). Nursing Home Reopening Recommendations Frequently Asked Questions. https://www.cms.gov/files/document/covid-nursing-home-reopening-recommendation-faqs.pdf.

[10] Carpenter J., Kooiman N., Kowalchik K., Mogle J., Newman J., Behrens L. (2022). Nursing Home Staff Risk Perceptions During the COVID-19 Pandemic. *Innovation in Aging*.

[11] Korstjens I., Moser A. (2018). Series: Practical Guidance to Qualitative Research. Part 4: Trustworthiness and Publishing. *The European Journal of General Practice*.

[12] Valerio M. A., Rodriguez N., Winkler P. (2016). Comparing Two Sampling Methods to Engage Hard-To-Reach Communities in Research Priority Setting. *BMC Medical Research Methodology*.

[13] Meertens R. M., Lion R. (2008). Measuring an Individual’s Tendency to Take Risks: The Risk Propensity Scale. *Journal of Applied Social Psychology*.

[14] Hsieh H. F., Shannon S. E. (2005). Three Approaches to Qualitative Content Analysis. *Qualitative Health Research*.

[15] Centers for Disease Control and Prevention (2020). Serious Infections and Outbreaks Occurring in LTCFs. https://www.cdc.gov/longtermcare/staff/report-publications.html.

[16] Bergman C., Stall N. M., Haimowitz D. (2020). Recommendations for Welcoming Back Nursing Home Visitors During the COVID-19 Pandemic: Results of a Delphi Panel. *Journal of the American Medical Directors Association*.

[17] Hugelius K., Harada N., Marutani M. (2021). Consequences of Visiting Restrictions During the COVID‐19 Pandemic: An Integrative Review. *International Journal of Nursing Studies*.

[18] Chan H. Y. L., Zhao Y. Y., Liu L., Chong Y. Y., Cheng H. Y., Chien W. T. (2022). Ethical Challenges Experienced by Care Home Staff During COVID-19 Pandemic. *Nursing Ethics*.

[19] Savage R. D., Rochon P. A., Na Y. (2022). Excess Mortality in Long-Term Care Residents With and Without Personal Contact With Family or Friends During the COVID-19 Pandemic. *Journal of the American Medical Directors Association*.

[20] Storr J., Kilpatrick C., Vassallo A. (2021). Safe Infection Prevention and Control Practices With Compassion—A Positive Legacy of COVID-19. *American Journal of Infection Control*.

[21] Lown B. A., Rosen J., Marttila J. (2011). An Agenda for Improving Compassionate Care: A Survey Shows About Half of Patients Say Such Care Is Missing. *Health Affairs*.

[22] Tehranineshat B., Rakhshan M., Torabizadeh C., Fararouei M. (2019). Compassionate Care in Healthcare Systems: A Systematic Review. *Journal of the National Medical Association*.

